# COVID-19 and SARS Coronavirus 2: Antibodies for the Immediate Rescue and Recovery Phase

**DOI:** 10.3389/fimmu.2020.01196

**Published:** 2020-05-29

**Authors:** Scott B. Halstead, Ramesh Akkina

**Affiliations:** ^1^Independent Researcher, Bethesda, MD, United States; ^2^Department of Microbiology, Immunology and Pathology, Colorado State University, Fort Collins, CO, United States

**Keywords:** antibody protection for Covid-19, SARS-CoV-2 immunity, gamma globulin for Covid-19 protection, antibody therapy for Covid-19, convalescent plasma for Covid-19 therapy, vaccine and therapeutics testing for Covid-19, human volunteer testing for Covid-19 protection, SARS Coronavirus 2 and Covid-19 immunity

The world is in the grip of a devastating SARS-CoV-2 pandemic causing a major health havoc and economic hardship/slowdown. In most affected countries mitigation of transmission by quarantining and social distancing is beginning to reduce hospitalization rates. However, current estimates are that the pandemic will continue for many months. What can be done immediately to control the damage and manage a transition to normalcy?

One approach is to reduce disease severity. A near-term possibility is to treat high risk patients with repurposed existing drugs (1). Another is to use antivirals such as remdesivir, a nucleotide analog, which has been previously shown to have efficacy against MERS disease in a monkey model (2, 3) and now under clinical investigation in China, USA and elsewhere. Recent preliminary results showed some efficacy and more in-depth studies are still underway (3). New compounds will undoubtedly emerge from the laboratory. Antibodies offer promising treatment options. Convalescent SARS antibodies administered early in acute illness were shown to reduce disease severity (4). Efforts are well-underway to manufacture therapeutic gamma globulin from COVID-19 convalescent sera, or alternatively to derive neutralizing monoclonal antibodies (5, 6).

A second approach is to protect high risk persons such as the elderly and persons with pre-existing conditions that include high blood pressure, diabetes, and obesity. Antibodies can be used to protect the vulnerable from infection. After WW II commercial gamma globulin was widely available affording short term protection against measles, paralytic poliomyelitis, hepatitis A, and hepatitis B (7–11). In the 1950s, a large scale blinded efficacy trial found that gamma globulin given to 100,000 children successfully blunted poliomyelitis attack rates (9). To prevent SARS-CoV-2 infections, gamma globulin antibody preparations or monoclonal antibodies can be given to those at high risk of fatal outcome. This requires use of another tool—epidemiology. Careful studies in populations suffering high infection rates should be able to identify risk factors for severe and fatal disease. Protective gamma globulin, once on the market, can be made available to self-identified high-risk persons through family health care providers. Persons in at high risk commercial occupations, health care workers and care givers should be protected. Commercial tests for detecting SARS-CoV-2 IgG antibodies are now on the market. Antibody testing can identify those who are immune and those who at risk and eligible for immunoprotection.

Progress is being made in developing neutralizing monoclonal human antibodies while at the same time the population of COVID-19 convalescents is growing rapidly. These antibodies should be put to work to help manage the pandemic. This will require that immune products be shown to prevent SARS-CoV-2 infections in human volunteers (12). SARS-CoV-2 has been adapted to grow in Vero cells (13). While there is risk, COVID-19 in young adults is seldom a severe disease. There is a long history of using a human challenge model to establish candidate therapeutic and preventive products for microbial pathogens (14–16). Such an approach should help shorten the typical long time it takes for vaccine/therapeutic testing. Once a protective level of antibody in humans can be correlated with an *in vitro* value it should be possible to screen candidate products more swiftly. The degree of protection may not confer complete sterilizing immunity but should impede viral spread to pulmonary stage and progression to severe disease. To avoid possible antibody-dependent enhancement (ADE) of COVID-19 infections, the Fc terminus of IgG antibodies should be removed or inactivated. However, this should be studied further to determine whether the risk of ADE outweighs the potential benefits afforded by antibody-dependent cellular cytotoxicity (ADCC) or antibody-dependent cellular phagocytosis (ADCP) (17, 18). Strategic exploitation of antibody-based approaches can help us return to normalcy. Indeed, as an example, using widespread serological testing, Germany is issuing “immunity certificates” to those who can safely re-enter the normal work force.

## Author Contributions

All authors listed have made a substantial, direct and intellectual contribution to the work, and approved it for publication.

## Conflict of Interest

The authors declare that the research was conducted in the absence of any commercial or financial relationships that could be construed as a potential conflict of interest.

## References

[1] Gao J Tian Z Yang X. Breakthrough: chloroquine phosphate has shown apparent efficacy in treatment of COVID-19 associated pneumonia in clinical studies. Biosci Trends. (2020) 14:72–3. 10.5582/bst.2020.01047 32074550

[2] de Wit E Feldmann F Cronin J Jordan R Okumura A Thomas T. Prophylactic and therapeutic remdesivir (GS-5734) treatment in the rhesus macaque model of MERS-CoV infection. Proc Natl Acad Sci USA. (2020) 117:6771–6. 10.1073/pnas.1922083117 32054787 PMC7104368

[3] Mahase E. Covid-19: Remdesivir is helpful but not a wonder drug, say researchers. BMJ. (2020) 369:m1798. 10.1136/bmj.m1798 32357949

[4] Mair-Jenkins J Saavedra-Campos M Baillie JK Cleary P Khaw FM Lim WS. The effectiveness of convalescent plasma and hyperimmune immunoglobulin for the treatment of severe acute respiratory infections of viral etiology: a systematic review and exploratory meta-analysis. J Infect Dis. (2015) 211:80–90. 10.1093/infdis/jiu396 25030060 PMC4264590

[5] Shanmugaraj B Siriwattananon K Wangkanont K Phoolcharoen W. Perspectives on monoclonal antibody therapy as potential therapeutic intervention for Coronavirus disease-19 (COVID-19). Asian Pac J Allergy Immunol. (2020) 38:10–8. 10.12932/AP-200220-0773 32134278

[6] Casadevall Pirofski L. The convalescent sera option for containing COVID-19. J Clin Invest. (2020) 130:1545–8. 10.1172/JCI138003 32167489 PMC7108922

[7] Young MK Nimmo GR Cripps AW Jones MA. Post-exposure passive immunisation for preventing measles. Cochrane Database Syst Rev. (2014) 1:Cd010056. 10.1002/14651858.CD010056.pub2 24687262 PMC11055624

[8] Janeway CA. Use of concentrated human serum gamma-globulin in the prevention and attenuation of measles. Bull N Y Acad Med. (1945) 21:202–22. 19312433 PMC1869384

[9] Rinaldo CR Jr. Passive immunization against poliomyelitis: the Hammon gamma globulin field trials, 1951-1953. Am J Public Health. (2005) 95:790–9. 10.2105/AJPH.2004.040790 15855454 PMC1449257

[10] Krugman S Ward R. Infectious hepatitis: current status of prevention with gamma globulin. Yale J Biol Med. (1961) 34:329–39. 14459922 PMC2605047

[11] Smetana HF Smetana FG. Viral hepatitis in United States soldiers stationed in Korea, 1967-1970: prophylactic efficacy of gamma globulin. Bull N Y Acad Med. (1976) 52:535–60. 58695 PMC1807201

[12] Eyal N Lipsitch M Smith PG. Human challenge studies to accelerate coronavirus vaccine licensure. J Infect Dis. (2020) 221:1752–6. 10.1093/infdis/jiaa152 32232474 PMC7184325

[13] Harcourt J Tamin A Lu X Kamili S Sakthivel SK Murray J. Severe acute respiratory syndrome coronavirus 2 from patient with (2019). novel coronavirus disease, United States. Emerg Infect Dis. (2020) 26:1266–73. 10.3201/eid2606.200516 32160149 PMC7258473

[14] Sabin AB Schlesinger RW. Production of immunity to dengue with virus modified by propagation in mice. Science. (1945) 101:640–2. 10.1126/science.101.2634.640 17844088

[15] Kirkpatrick BD Whitehead SS Pierce KK Tibery CM Grier PL Hynes NA. The live attenuated dengue vaccine TV003 elicits complete protection against dengue in a human challenge model. Sci Transl Med. (2016) 8:330ra36. 10.1126/scitranslmed.aaf1517 27089205

[16] Matuschewski K Borrmann S. Controlled Human Malaria Infection (CHMI) Studies: Over 100 Years of Experience with Parasite Injections. Methods Mol Biol. (2019) 2013:91–101. 10.1007/978-1-4939-9550-9_7 31267496

[17] Wan Y Shang J Sun S Tai W Chen J Geng Q. Molecular mechanism for antibody-dependent enhancement of coronavirus entry. J Virol. (2020) 94:e2015–19. 10.1128/JVI.02015-19 31826992 PMC7022351

[18] Halstead SB Mahalingam S Marovich MA Ubol S Mosser DM. Intrinsic antibody-dependent enhancement of microbial infection in macrophages: disease regulation by immune complexes. Lancet Infect Dis. (2010) 10:712–22. 10.1016/S1473-3099(10)70166-3 20883967 PMC3057165

